# Trends on detecting malignant skin neoplasms during the National Campaigns of Skin Cancer Prevention (2000‒2023)^☆^

**DOI:** 10.1016/j.abd.2024.04.002

**Published:** 2024-06-08

**Authors:** Aline Guimarães Grana, Heitor de Sá Gonçalves, Carlos Baptista Barcaui, Carolina Talhari, Hélio Amante Miot

**Affiliations:** a Postgraduate Program in Sciences Applied to Dermatology, Universidade do Estado do Amazonas, Manaus, AM, Brazil; b Centro de Referência Nacional em Dermatologia Sanitária Dona Libânia, Secretaria da Saúde, Governo do Estado do Ceará, Fortaleza, CE, Brazil; c Instituto de Dermatologia Prof. Rubem David Azulay, Santa Casa da Misericórdia do Rio de Janeiro, Rio de Janeiro, RJ, Brazil; d Department of Infectology, Dermatology, Imaging Diagnosis and Radiotherapy, Faculty of Medicine, Universidade Estadual Paulista, Botucatu, SP, Brazil

Dear Editor,

The National Skin Cancer Prevention Campaign (CNPCP) is an event that has taken place annually since 1999, aimed at education and fighting the disease by disseminating information, and promoting health education, diagnosis and quality treatment free of charge to the population. On a single summer day, there is the in-person examination of thousands of individuals. The CNPCP is integrated into the “Orange December” project, coordinated by the Brazilian Society of Dermatology (SBD, *Sociedade Brasileira de Dermatologia*), with the participation of accredited services and associates from all over Brazil.^1^

According to SBD bulletins, since its first edition, the CNPCP has carried out the examination of more than 600,000 individuals and led to the suspected diagnosis of more than 75,000 malignant neoplastic lesions of the skin, including basal cell carcinomas (BCC), squamous cell carcinomas (SCC) and melanomas. This is the largest worldwide action aimed at screening for malignant skin neoplasms, surpassing the initiatives of the European community.^2^ In addition to actions associated with population health education, encouraging self-examination and prevention (primary and secondary), the CNPCP generates information that can support public policies, and professional development, in addition to guiding the training of specialists and sizing the dermatological care force.

This study aimed to describe the main data and evaluate aspects associated with trends in detecting malignant cutaneous neoplasms during CNPCPs.

Data from the CNPCP participants were obtained from SBD, which were summarized nationally and analyzed according to the year of the campaign (2000 to 2023). The time series was analyzed according to autoregressive integrated moving average (ARIMA) models, with imputation for missing data. The correlation between the variables was estimated by Pearson’s correlation coefficient (r), which varies from -1 to +1, with the sign indicating a positive or negative direction of the correlation between the variables, and the value suggesting the size of this correlation.^3^ A p-value < 0.05 was considered significant.

Table 1 depicts the main summarized data from the campaigns. The number of consultations showed a peak, followed by a gradual reduction from 2006 onwards (Fig. 1). The proportion of suspected cases of malignant skin neoplasms, among the attended cases, increased depending on the years (Fig. 2), from 7.7% (2000) to 29.3% of the consultations (2023).

**Table 1:** Main data regarding skin cancer campaigns (2000 to 2023).

Year	Consultations	Males	White-skinned	Brown- skinned	Black- skinned	Unprotected sun exposure	Previous SC	Family SC	Suspected BCC	Suspected SCC	Suspected MM	Suspected SC
2000	24,500	NA	NA	NA	NA	NA	NA	NA	5.8%	0.8%	0.5%	7.7%
2001	32,007	35.2%	57.2%	29.8%	6.4%	61.5%	5.3%	12.2%	5.8%	1.0%	0.5%	7.9%
2002	27,758	NA	NA	NA	NA	69.0%	NA	NA	6.5%	1.5%	0.5%	8.6%
2003	37,853	37.9%	62.7%	29.6%	6.8%	69.6%	7.1%	14.4%	6.5%	1.2%	0.6%	8.6%
2004	33,682	40.0%	65.1%	27.3%	6.5%	69.4%	NA	NA	6.3%	1.3%	0.5%	8.4%
2005	34,928	NA	NA	NA	6.5%	68.5%	NA	NA	7.0%	1.5%	0.7%	8.7%
2006	41,751	NA	NA	NA	NA	67.6%	NA	NA	NA	NA	NA	9.5%
2007	31,429	37.5%	NA	NA	NA	67.7%	8.1%	16.6%	NA	NA	0.6%	10.0%
2009	35,019	39.2%	61.4%	30.9%	6.8%	64.5%	9.1%	17.8%	8.2%	1.9%	0.9%	11.6%
2010	32,376	37.6%	62.9%	29.0%	7.0%	63.3%	9.2%	18.5%	8.4%	1.9%	0.9%	11.7%
2011	31,697	39.2%	64.8%	27.9%	6.0%	61.4%	11.3%	20.3%	9.3%	2.2%	1.0%	13.2%
2012	33,259	39.4%	63.8%	28.9%	6.0%	62.9%	11.5%	21.4%	9.9%	2.5%	1.1%	14.3%
2013	28,895	38.1%	62.1%	30.4%	5.8%	61.0%	11.0%	22.5%	9.4%	2.2%	1.2%	13.5%
2014	24,727	39.1%	60.7%	31.4%	6.2%	59.9%	12.0%	23.0%	10.1%	2.4%	1.1%	14.3%
2015	24,092	40.7%	NA	58.8%	6.9%	63.0%	11.0%	22.8%	10.1%	2.6%	1.3%	14.7%
2016	28,132	39.6%	36.3%	52.5%	7.2%	63.7%	12.5%	23.1%	10.0%	2.9%	1.4%	15.1%
2017	26,480	39.2%	33.8%	53.9%	8.1%	63.6%	12.2%	24.2%	10.5%	2.8%	1.4%	15.5%
2018	25,006	39.8%	36.9%	52.4%	6.7%	62.2%	14.4%	24.9%	11.0%	3.4%	1.4%	16.8%
2019	23,227	40.6%	37.8%	55.4%	6.9%	62.9%	13.9%	24.7%	12.1%	3.7%	1.8%	18.5%
2022	11,604	39.3%	37.3%	56.2%	6.4%	63.3%	15.2%	27.4%	15.2%	4.6%	2.3%	23.5%
2023	15,967	45.8%	45.8%	62.8%	8.4%	73.9%	18.9%	31.3%	18.8%	6.1%	2.8%	29.3%

NA, Not available; SC, Skin Cancer; BCC, Basal Cell Carcinoma; SCC, Squamous Cell Carcinoma; MM, Melanoma.

**Figure 1:**
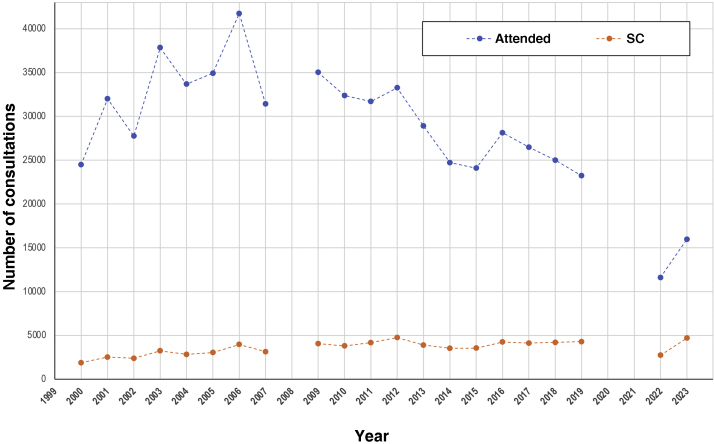
Time series of the number of consultations and the number of suspected skin cancer (SC) cases, among those who attended the national skin cancer prevention campaigns (2000‒2023). Note: Data from the 2008 campaign are not available, and there were no consultations in the years 2020 and 2021, due to the contingency of the COVID-19 pandemic.

**Figure 2:**
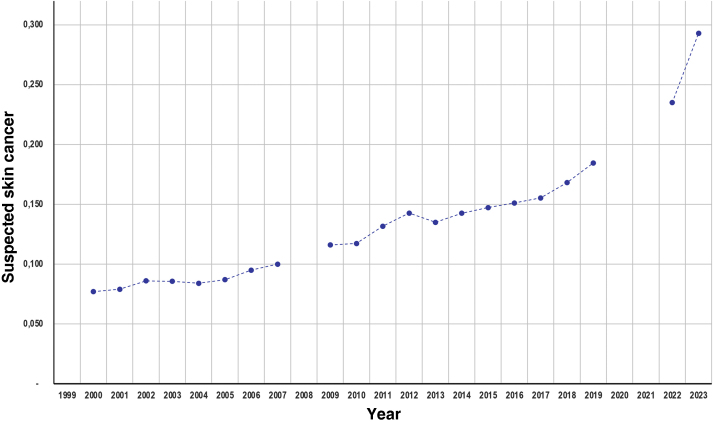
Rates of suspected skin cancer (basal cell carcinoma, squamous cell carcinoma and melanoma), among those who attended the national skin cancer prevention campaigns (2000‒2023). Note: Data from the 2008 campaign are not available, and there were no consultations in the years 2020 and 2021, due to the contingency of the COVID-19 pandemic.

The average annual linear increase was 0.53% (0.47%‒0.58%) regarding the rate of suspected malignant skin neoplasms until 2019 (p < 0.01). However, the figures for 2022 and 2023 were higher than this projection. According to the time series model, the national prediction (95% CI) of suspected malignant cutaneous neoplasms for the 2024 CNPCP is 32.7% (30.4%‒35.1%) of consultations (R^2^ = 0.96).

When evaluating the correlations between the rates of suspected malignant neoplasms of the skin and the other covariates, the proportion of consultations for males, brown-skinned individuals, people with a personal history of skin cancer, and those with a family history of skin cancer were positively correlated with the outcome (p < 0.01). Whereas, the proportion of individuals who do not use photoprotection did not correlate with the rate of suspected malignant cutaneous neoplasms in this time series (Fig. 3).

**Figure 3:**
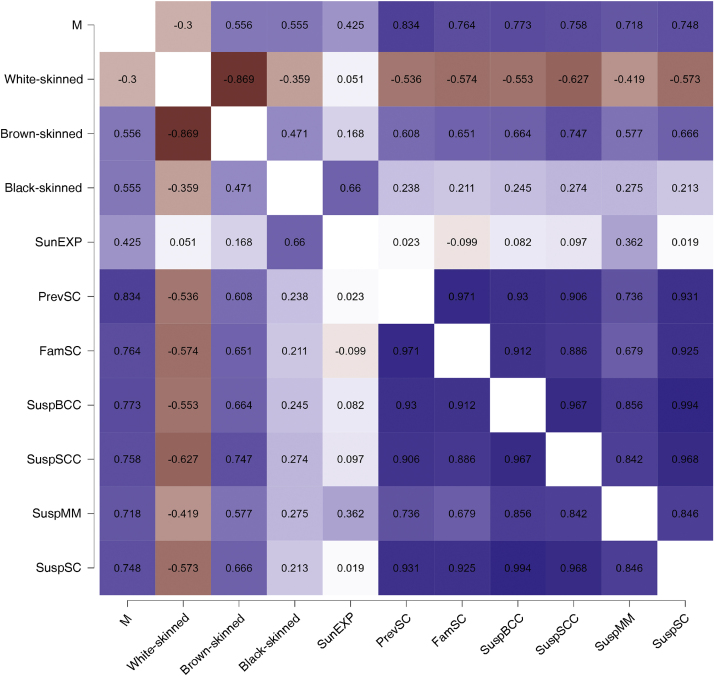
Heat map with Pearson correlation coefficients related to the study covariates. Caption: M, Males; SunEXP, Unprotected Sun Exposure; PrevSC, Personal History of Skin Cancer; FamSC, Family History of Skin Cancer; SuspBCC, Suspected Basal Cell Carcinoma; SuspSCC, Suspected Squamous Cell Carcinoma; SuspMM, Suspected Melanoma; SuspSC, Suspected Skin Cancer.

Individually, the rates of suspected malignant skin neoplasms (BCC, SCC and melanoma) increased proportionally in the period and showed a high correlation (r > 0.8) with each other (Fig. 3). The prediction of suspected neoplasms for the 2024 CNPCP is 20.1% (16.1%‒24.7%) for BCC; 6.2% (5.0%‒7.4%) for SCC; and 3.1% (2.0%‒4.0%) for melanoma.

The increasing rate of suspected malignant skin neoplasms in the CNPCP may reflect several aspects, such as the effect of raising risk awareness among the participants (effective health education), population aging, increased incidence of skin cancer, increased solar radiation, and the abbreviated search for resolution given the delay in scheduling dermatological consultations in the public health system.^4^, ^5^, ^6^ Improving data collection in future campaigns would be important and could eventually assess the causes underlying this phenomenon.

Knowledge about skin cancer and adherence to photoprotection strategies have the potential to reduce the incidence and morbidity and mortality of skin cancer, with potential cost reduction for the health system. In addition to the different morphologies of skin cancer cases, the ethnic, climatic and sun exposure pattern diversity that characterizes the reality of the Brazilian population poses challenges in the development of unique education strategies. Even among educated populations, in higher-risk regions, such as university students in Rio Grande do Sul, there is low adherence to daily photoprotection, high frequency of sunburns, and still insufficient knowledge about photoeducation.^7^

Climate changes such as reduced cloud cover, as well as ozone layer depletion and increased solar activity are cofactors known to be associated with higher ultraviolet radiation on the planet surface, which is fundamental in skin carcinogenesis.^8^, ^9^ In the albino community of Malawi, heat waves caused by climate change have been associated with skin cancer risk, which causes 90% of all deaths in these individuals. In that country, life expectancy is 65 years; however, in the albino community, it is less than 30 years.^10^

CNPCP has an important effect on the early identification of malignant cutaneous neoplasms in at-risk individuals. In a tertiary public service, in the interior of the state of São Paulo, the BCC cases identified by the CNPCP were smaller in diameter than those referred through the public health system, an observation more significant in males.^11^ However, the difficulty of public services in the surgical resolution of cases identified in the campaign is an institutional challenge for their adherence to the campaign.

The limitations of the present study are based on the quality of the information provided by dermatologists, the lack of information associated with the age range of care, the lack of detailed data regarding the 2008 campaign, the lack of diagnostic confirmation of suspected cases, the sampling of large urban centers only, where the campaigns take place, and in the absence of the event during the years of the COVID-19 pandemic (2020 and 2021). Although the CNPCP occurs nationwide, Brazil has geoclimatic and ethnic variations, as well as very regionalized health policies. Therefore, the results of the campaigns may show regional particularities that were not the target of this study.

In conclusion, there was a gradual increase in the rate of suspected diagnoses of cutaneous neoplasms during CNPCPs in the studied period. The “Orange December” actions, including the CNPCP, are important for the promotion of public health in dermatology.

## Financial support

None declared.

## Authors’ contributions

Aline Guimarães Grana: Design and planning of the study; analysis and interpretation of data; statistical analysis; drafting and editing of the manuscript; critical review of the literature; critical review of the manuscript; approval of the final version of the manuscript.

Heitor de Sá Gonçalves: Drafting and editing of the manuscript; critical review of the literature; critical review of the manuscript; approval of the final version of the manuscript.

Carlos Baptista Barcaui: Drafting and editing of the manuscript; critical review of the literature; critical review of the manuscript; approval of the final version of the manuscript.

Carolina Talhari: Design and planning of the study; analysis and interpretation of data; statistical analysis; drafting and editing of the manuscript; critical review of the literature; critical review of the manuscript; approval of the final version of the manuscript.

Hélio Amante Miot: Design and planning of the study; analysis and interpretation of data; statistical analysis; drafting and editing of the manuscript; critical review of the literature; critical review of the manuscript; approval of the final version of the manuscript.

## Conflicts of interest

None declared.
